# Genome-wide analysis of RNAs associated with *Populus euphratica* Oliv. heterophyll morphogenesis

**DOI:** 10.1038/s41598-018-35371-x

**Published:** 2018-11-22

**Authors:** Shao-Wei Qin, Ren-Jun Jiang, Na Zhang, Zhan-Wen Liu, Cai-Lin Li, Zhong-Zhong Guo, Liang-Hong Bao, Li-Feng Zhao

**Affiliations:** 1 College of Life Sciences, Tarim University, Alar, 843300 China; 2 Key Laboratory of Protection and Utilization of Biological Resources in Tarim Basin, Tarim University, Alar, 843300 China

## Abstract

The desert plant *Populus euphratica* Oliv. has typical heterophylly; linear (Li), lanceolate (La), ovate (Ov) and broad-ovate (Bo) leaves grow in turn as trees develop to maturity. *P*. *euphratica* is therefore a potential model organism for leaf development. To investigate the roles of RNAs (including mRNAs, miRNAs, lncRNAs and circRNAs) in the morphogenesis of *P*. *euphratica* heterophylls, juvenile heterophylls were sampled individually, and then, the expression patterns of miRNAs, mRNAs, lncRNAs and circRNAs were analysed by small RNA sequencing and strand-specific RNA sequencing. We found that 1374 mRNAs, 19 miRNAs, 71 lncRNAs and 2 circRNAs were *P*. *euphratica* heterophyll morphogenesis–associated (PHMA) RNAs; among them, 17 PHMA miRNAs could alter the expression of 46 PHMA mRNAs. Furthermore, 11 lncRNAs and 2 circRNAs interacted with 27 PHMA mRNAs according to the ceRNA hypothesis. According to GO and KEGG pathway analysis, PHMA RNAs were mainly involved in metabolism, response to stimulus and developmental processes. Our results indicated that external environmental factors and genetic factors in *P*. *euphratica* co-regulated the expression of PHMA RNAs, repressed cell division, reinforced cell growth, and ultimately resulted in the morphogenesis of *P*. *euphratica* heterophylls.

## Introduction

Generally, the shapes of leaves are determined by variations in development along their three axes, *i*.*e*., the adaxial-abaxial, apical-basal and medial-lateral axes^1–3^, and this process is co-regulated by genes and environmental factors^4^. In most plants, changes in leaf shape during different developmental stages are slight, but in the desert- and drought-endemic plant *Populus euphratica* Oliv., these changes are very clear. During the germination and seedling stages, *P*. *euphratica* leaves are linear (Li), having a leaf index (LI, leaf length/leaf width) ≥5. Then, during the development of the tree, the leaves become lanceolate (La, 5 > LI ≥ 2), ovate (Ov, 2 > LI ≥ 1) and broad-ovate (Bo, LI < 1) in turn^5,6^. This heterophylly makes *P*. *euphratica* a potential model organism for studying leaf development.

Non-coding RNAs (ncRNAs) mainly consist of microRNAs (miRNAs), long non-coding RNAs (lncRNAs) and circular RNAs (circRNAs), and the latter two can be regarded as sponges that decoy miRNAs and play vital roles in the development of animals and plants^7–9^. However, knowledge of the roles of lncRNAs and circRNAs in leaf development remains scarce at present.

Previous studies have found that the shapes of *P*. *euphratica* heterophylls underwent only minor changes after unfolding, and the shapes of the leaves from the same bud were similar for all leaf shapes; furthermore, the development of *P*. *euphratica* heterophylls during leaf germination could be divided into early (EA), middle (MI), and late (LA) periods^5,10^. In all stages, the macroscopic juvenile leaf second from the shoot apical meristem in each bud represented the middle period of *P*. *euphratica* heterophyll genesis that growing was fastest^5^. To understand the roles of RNAs in *P*. *euphratica* heterophyll morphogenesis, the categories and expression patterns of mRNAs, miRNAs, lncRNAs and circRNAs in Li, La, Ov and Bo leaves were investigated by sequencing. Their interactions and networks were constructed, and their functions in *P*. *euphratica* heterophyll morphogenesis were further predicted.

## Results

### Leaf length, leaf width and leaf index of *P. euphratica* heterophylls

**Figure 1:**
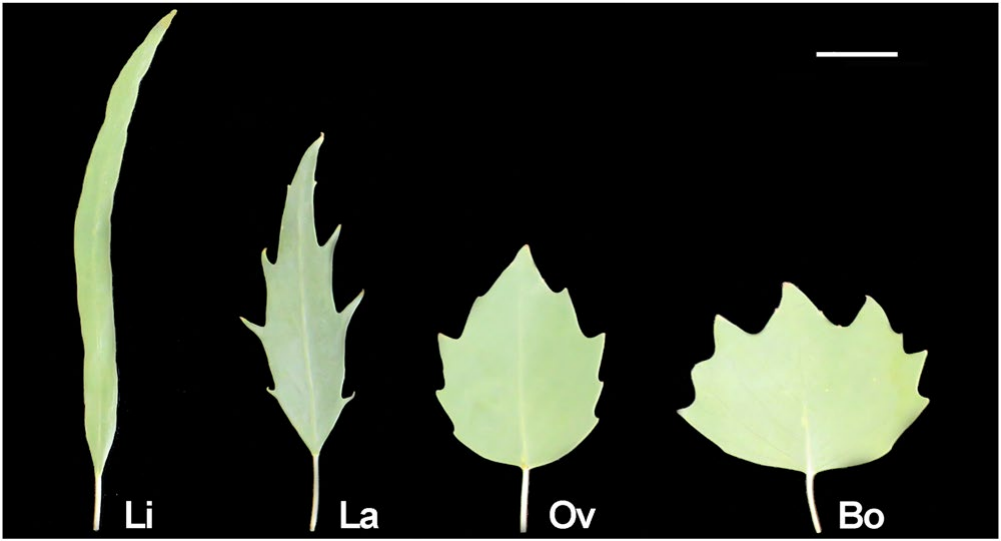
Leaf shapes from representative buds of the 4 groups of *P*. *euphratica* heterophyll samples. The scale bar shows 1 cm.

Three biological replicates were measured for the Li, La, Ov and Bo groups (Fig. 1): Li1, Li2, Li3, La1, La2, La3, Ov1, Ov2, Ov3, Bo1, Bo2 and Bo3. The average values of leaf length (L), leaf width (W) and LI, which were obtained from 12 samples at the early stage of leaf expansion, are shown in Table 1. In general, L decreased slowly from Li to Bo leaves; however, this rule could be broken in some cases. W increased from Li to Bo. In contrast to W, LI decreased obviously from Li (7.45) to Bo (0.73) (approximately 10-fold). Unlike L, W and LI showed regular change trends.

**Table 1:** Leaf shape data from 12 *P*. *euphratica* samples.

Items	Li1	Li2	Li3	La1	La2	La3	Ov1	Ov2	Ov3	Bo1	Bo2	Bo3
L (cm)	2.37	3.56	2.52	1.96	2.55	2.65	2.19	2.17	2.66	1.19	1.31	1.30
W (cm)	0.36	0.50	0.37	0.86	0.75	0.82	1.36	1.51	1.28	1.63	1.84	1.80
LI (ratio)	6.78	7.45	7.10	2.68	4.05	3.39	1.78	1.58	2.07	0.74	0.73	0.74

L, W and LI represent leaf length, leaf width, and leaf index, respectively.

### Expression patterns of RNAs in *P. euphratica* heterophyll morphogenesis

In this study, 258 miRNAs, including 167 annotated miRNAs and 91 novel miRNAs (Table 2), were obtained from small RNA sequencing of 12 samples. A total of 3312 lncRNAs (2870 annotated and 442 novel lncRNAs) were obtained from the filtered strand-specific RNA sequencing results (Table 2). A total of 1149 circRNAs were predicted from the sequences of the 12 samples (Table 2).

**Table 2:** Counts of miRNAs, lncRNAs and circRNAs in *P*. *euphratica* heterophyll samples.

Samples	Li1	Li2	Li3	La1	La2	La3	Ov1	Ov2	Ov3	Bo1	Bo2	Bo3	Total
Annotated miRNAs	124	129	130	132	120	133	139	124	123	113	125	115	167
Novel miRNAs	64	70	67	71	65	65	72	68	67	70	66	75	91
Annotated lncRNAs	2241	2179	2130	2216	2187	2179	2191	2215	2253	2220	2238	2217	2870
Novel lncRNAs	400	383	411	386	390	383	397	396	393	392	399	389	442
Novel circRNAs	178	153	187	224	184	281	240	205	280	176	188	182	1149

**Figure 2:**
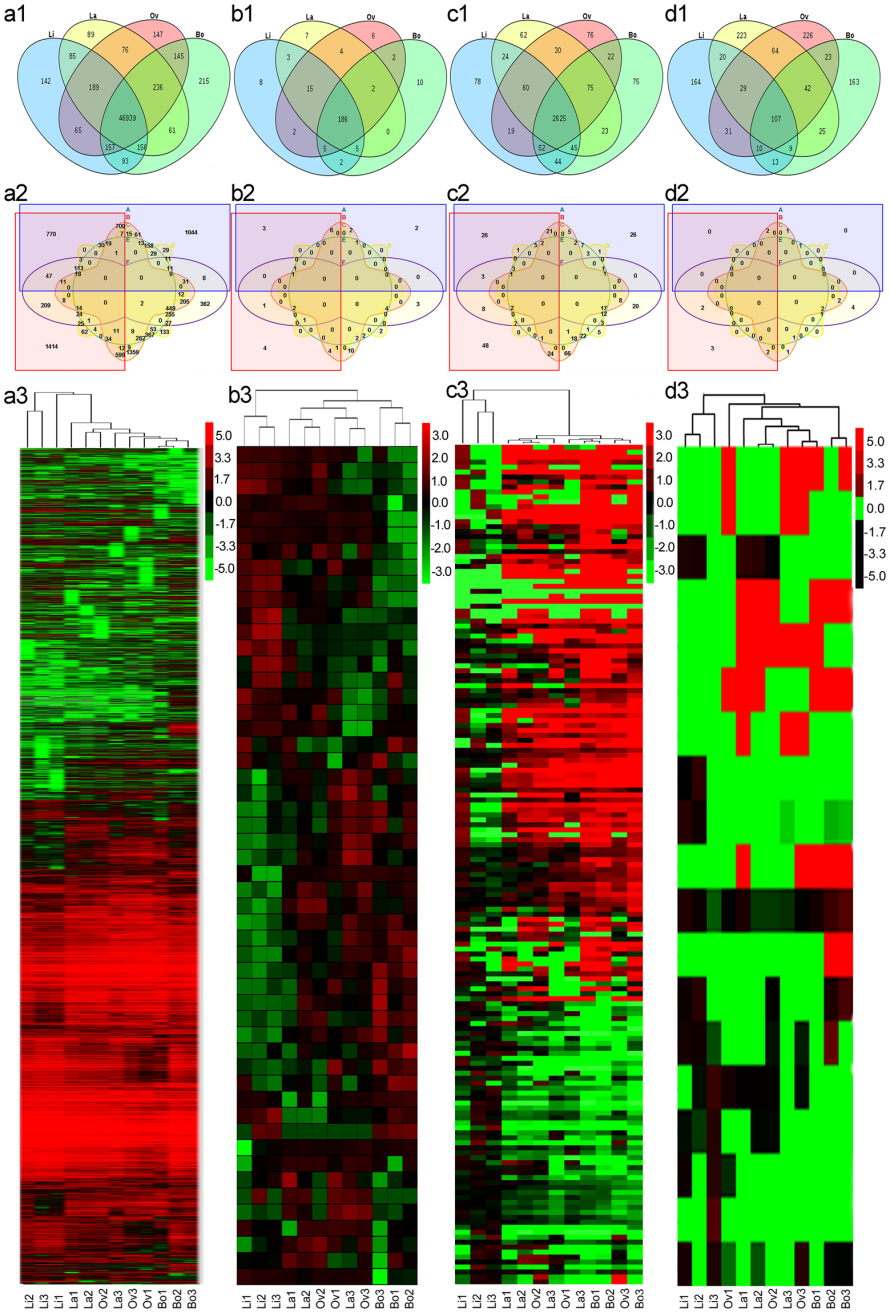
Venn diagrams and cluster analyses of differentially expressed (DE) RNAs. (**a**–**d**) display the DE mRNAs, miRNAs, lncRNAs and circRNAs, respectively. For (**a1**–**d1**), each number represents the count of expressed RNAs in certain leaf types. For (**a2**–**d2**), each number represents the count of DE RNAs between certain group pairs. Among them, A, B, C, D, E and F represent La/Li, Ov/Li, Bo/Li, Ov/La, Bo/La and Bo/Ov, respectively. For (**a3**–**d3**), they display cluster analyses of RNAs. The up- and downregulated fold changes (log_2_) of RNAs are coloured according to each panel key.

The statistical analysis showed that some genes were expressed in only a certain leaf type, while the others were expressed in several kinds of leaves (Fig. 2a1–d1). For example, 142, 89, 147 and 215 mRNAs were expressed in only Li, La, Ov and Bo leaves, respectively (Fig. 2a1). To identify the differentially expressed (DE) RNAs among the 4 groups of leaves, the transcriptomes of six group pairs were compared: La/Li (A), Ov/Li (B), Bo/Li (C), Ov/La (D), Bo/La (E) and Bo/Ov (F) (Fig. 2a2–d2). The number of DE mRNAs was 8942 (*P* < 0.05), and among them, there were 3147, 4145, 3899, 455, 1906 and 1885 DE mRNAs in A, B, C, D, E and F, respectively (Fig. 2a2).

The cluster maps of DE RNAs (4 groups, 12 samples) are displayed in Fig. 2a3–d3. For mRNAs and miRNAs, the difference between the Li and Bo groups was clear, while La and Ov overlapped (Fig. 2a3,b3). For lncRNAs, only the Li group differed from the other groups, while the La, Ov and Bo groups overlapped (Fig. 2c3). For circRNAs, the difference between the Li and Bo groups was strong, while La and Ov overlapped (Fig. 2d3).

### *P. euphratica* heterophyll morphogenesis–associated RNAs

**Figure 3:**
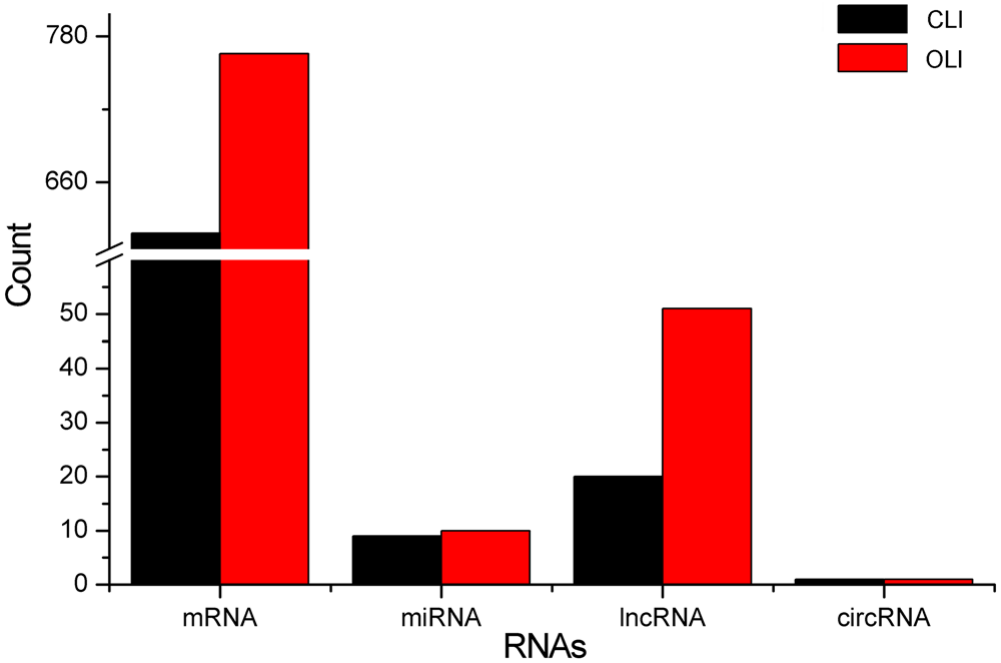
Counts of RNAs associated with *P*. *euphratica* heterophyll morphogenesis. CLI and OLI indicate expression change trends corresponding to and opposite the changes in LI, respectively.

If the expression pattern of an RNA was completely consistent with the LI changes (CLI) or opposite the LI changes (OLI), and there was a significant difference between Li and Bo, that RNA was considered a *P*. *euphratica* heterophyll morphogenesis–associated (PHMA) RNA. Counts of PHMA RNAs are displayed in Fig. 3. Of these, 1374 mRNAs were PHMA, of which 607 mRNAs were CLI, and 767 mRNAs were OLI. For miRNAs, the counts were 19, 9 and 10, respectively. For lncRNAs, the counts were 71, 20 and 51, respectively. For circRNAs, the counts were 2, 1 and 1, respectively. Additionally, the expression profiles of PHMA mRNAs, miRNAs, lncRNAs and circRNAs are shown in Supplementary Datasets D1–D4, respectively.

### qPCR validation

**Figure 4:**
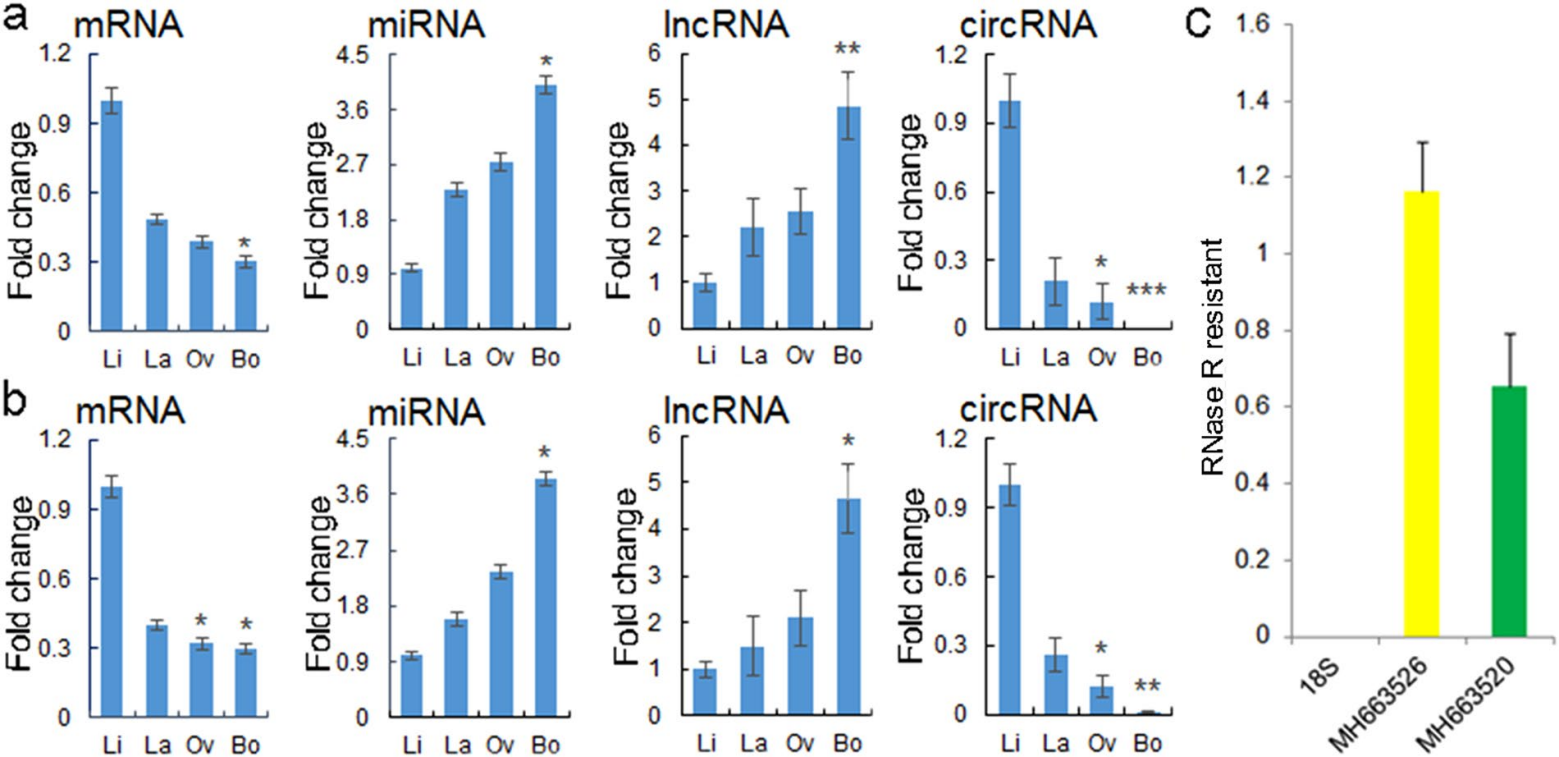
qPCR validation and RNase R resistance test. (**a**) Sequencing results. *P < 0.05, **P < 0.01, and ***P < 0.001 represent the significance of the comparison of each sample with Li leaves by DESeq. (**b**) qPCR results of corresponding RNAs. *P < 0.05 and **P < 0.01 represent the significance of the comparison of each sample with Li leaves by Student’s *t* test. (**c**) RNase R resistance test result. 18S was used as a linear control gene. The Y axis indicates the ratio of RNase R treatment/RNase R-free treatment; the X axis indicates the gene symbol or ID. Error bars indicate ± SD.

The sequencing expression profiles of 4 randomly selected RNAs, the mRNA XM_011028982.1, miRNA MH663522, lncRNA XR_843201.1 and circRNA MH663520, are shown in Fig. 4a. The expression levels of the corresponding RNAs by quantitative reverse transcription polymerase chain reaction (qPCR) are displayed in Fig. 4b. The results clearly show that all the validated RNA profiles were consistent with the results obtained from sequencing. Furthermore, the results of circRNA RNase resistance experiments indicated that MH663526 could resist RNase R digestion completely, MH663520 could only partly resist RNase R, while 18 S could not resist digestion at all (Fig. 4c).

### Regulatory networks and functional predictions of PHMA RNAs

**Figure 5:**
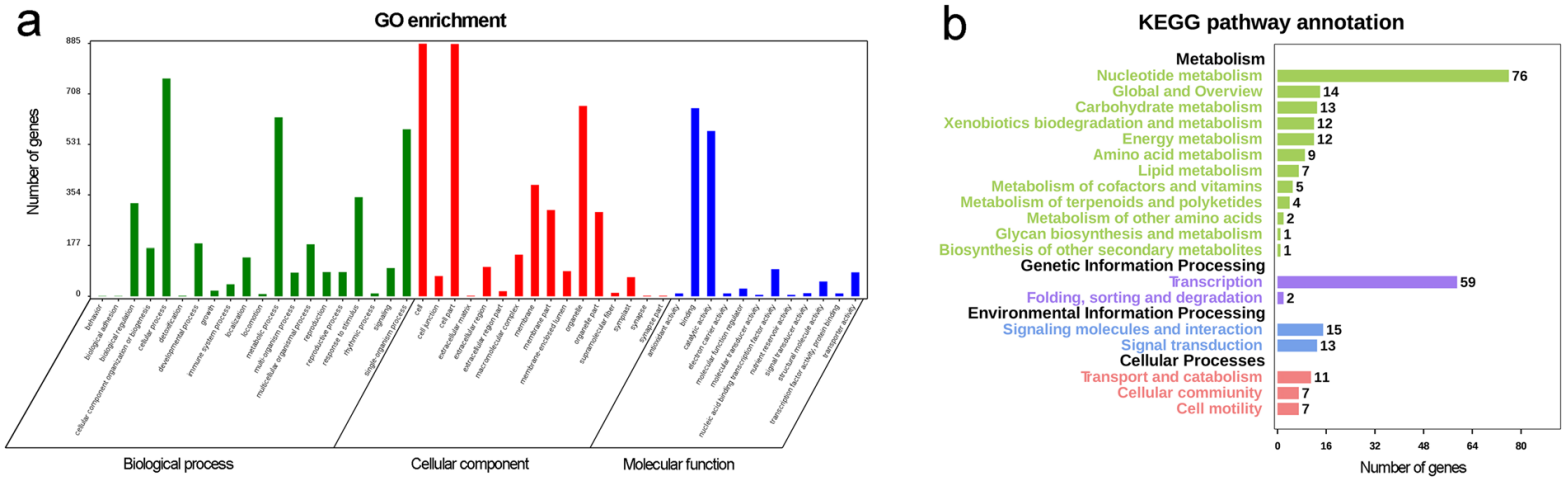
GO and KEGG pathway enrichment of PHMA mRNAs. (**a**) GO enrichment; (**b**) KEGG pathway enrichment.

According to GO, 1168 of 1374 PHMA mRNAs could be annotated, and these mRNAs were associated with 930 GO terms (D1). The number of PHMA mRNAs related to biological process, cellular component and molecular function are displayed in Fig. 5a. Notably, 626 PHMA mRNAs participated in metabolic processes, 346 PHMA mRNAs were associated with response to stimulus, and 184 PHMA mRNAs were related to the development process in the biological process category. The number of mRNAs annotated in KEGG was 343 (D1). According to the KEGG enrichment, the products of PHMA mRNAs were mainly involved in 4 pathways, metabolism, genetic information processing, environmental information processing and cellular processes (Fig. 5b). Additionally, the GO and KEGG annotations of all PHMA mRNAs are also shown in D1.

**Figure 6:**
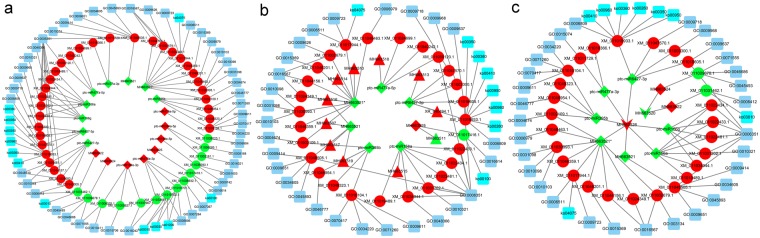
Regulatory networks and functional predictions of ncRNAs and targets. The dark blue squares represent GO IDs; the turquoise blue squares represent KEGG pathway IDs; the diamonds, triangles, Vs and ellipses represent miRNAs, lncRNAs, circRNAs and mRNAs, respectively. For the RNAs, red indicates OLI, and green indicates CLI. (**a**) Regulatory networks of miRNAs. (**b**) regulatory networks of lncRNAs. (**c**) Regulatory networks of circRNAs.

Target prediction and expression correction analysis showed that 17 PHMA miRNAs could regulate 46 PHMA mRNAs during leaf development (Fig. 6a). Representative GO terms and all KEGG pathways are displayed in Fig. 6a. For example, MH663525 was OLI, which caused its target (XM_011017416.1) to be CLI. According to the GO and KEGG annotations, MH663525 regulated targets in the steroid biosynthesis pathway (ko00100) and the oxidoreductase process (GO:0016614).

The competing endogenous RNA (ceRNA) hypothesis suggested that competing endogenous RNAs could serve as miRNA sponges and thereby impair the activity of miRNAs mediating gene silencing, thus protecting those mRNAs that shared miRNAs with them^7^. Based on the lncRNA-mRNA pairs (sharing miRNAs) and expression profiles, it was found that 11 PHMA lncRNAs could decoy 7 PHMA miRNAs and regulate 27 mRNAs. These 11 lncRNAs were mainly involved in 36 biological processes (GO) and 8 pathways (KEGG) by regulating their targets (Fig. 6b). For example, both XR_840494.1 and MH663513 were OLI; as targets of ptc-miR6427-3p, they could decoy ptc-miR6427-3p, causing the latter to be CLI, and their targets (XM_011003300.1, XM_011016605.1, XM_011019933.1 and XM_011047570.1) were OLI. According to GO and KEGG, XR_840494.1 and MH663513 were mainly involved in the anthocyanin-containing compound biosynthetic process (GO:0009718), glycine, serine and threonine metabolism (ko00260) and other pathways by regulating their targets (Fig. 6b).

Based on the circRNA-mRNA pairs (sharing miRNAs) and expression profiles, 2 PHMA circRNAs could decoy 9 miRNAs and regulate 27 mRNAs. According to GO and KEGG, 2 PHMA circRNAs were mainly involved in 37 biological processes and 9 pathways (Fig. 6c). For example, MH663526 was OLI and caused ptc-miR395b to be CLI, and their targets (XM_011002109.1, XM_011010104.1, XM_011046954.1, XM_011049323.1) were all OLI. According to GO and KEGG, MH663526 was mainly involved in cellular response to cold (GO:0070417) and other pathway by regulating these 4 targets (Fig. 6c).

## Discussion

*P*. *euphratica* leaves are linear, lanceolate, ovate and broad-ovate in turn as trees grow from juvenile to adult^5,6^, and this characteristic reflects a continually decreasing LI (Fig. 1 and Table 1). We found that thousands of *P*. *euphratica* DE RNAs participated in *P*. *euphratica* heterophyll morphogenesis. A cluster analysis of these DE RNAs implied that the expression patterns in each group were similar for all 4 classes of RNAs. Generally, the Li and Bo groups were located at the two extremes, while La and Ov overlapped (Fig. 2a3–d3). This result was related to the data of leaf shape (Table 1). The expression patterns of 637 RNAs were CLI (Fig. 3), indicating that these RNAs could prompt leaf elongation or inhibit broadening; the expression patterns of 829 RNAs were OLI (Fig. 3), suggesting that these RNAs could provoke the development of the medial-lateral axis or inhibit the development of the apical-basal axis. All these RNAs were referred to as PHMA RNAs.

Most PHMA RNAs were related to metabolic processes (Fig. 6). Kalve *et al*.^11,12^ illustrated that cell growth depended on metabolism; the cell expansion rate in the medial-lateral axis was higher than that in the apical-basal axis in the early period of leaf development. Our study indicated that metabolic processes could impact the development of leaves via additional pathways. For example, 18 PHMA mRNAs were involved in the starch and sucrose metabolism pathways; among them, 15 PHMA mRNAs were OLI, and 3 PHMA mRNAs were CLI (D1). These results indicated that saccharide metabolism was more active, and according to previously reported results, the content of soluble sugar in Bo leaves was higher, than that in Li leaves^10^. Yu *et al*.^13^ found that sugar could repress miR156 expression, and the latter plays a key role in leaf development^14^, so these 18 PHMA mRNAs could also affect leaf development by regulating the miR156 pathway.

The morphogenesis of *P*. *euphratica* heterophylls is closely related to environmental factors. For example, adult *P*. *euphratica* inhabiting riverbanks had more lanceolate leaves than those in deserts did^10^. Previous studies have found that both temperature and light can affect leaf shape^15,16^. In this study, it was found that 17 PHMA mRNAs were associated with response to cold, and 12 PHMA mRNAs were involved in response to light (D1). Furthermore, our results demonstrated that salt stress and oxidative stress could affect leaf development. For instance, XM_011017944.1, XM_011002679.1, XM_011048463.1 and XM_011030993.1 were co-regulated by MH663521*, MH663514, MH663516 and MH663510 (Fig. 6b). According to GO, the first three PHMA mRNAs were involved in response to oxidative stress (D1); the latter was involved in leaf development^17,18^. Based on the ceRNA hypothesis^7^, these RNAs could interact with each other, meaning that the first three genes could also affect leaf development. Thus, environmental factors might play a critical role in *P*. *euphratica* heterophyll morphogenesis by regulating PHMA RNAs involved in responses to stimuli.

Development, including polarity establishment, cell proliferation and division, expansion and growth, along the three axes determines leaf shape in plants^15^. When the leaf length was shorter than 2 mm, the LI of *P*. *euphratica* heterophylls was similar, but later, differences in development along the apical-basal and medial-lateral axes caused heterophylly^5^. Only 3 PHMA RNAs were found to be involved in leaf polarity establishment. The axial regulators *yabby1* (XM_011019167.1) and *yabby5-like* (XM_011021842.1) were CLI (D1), and the *yabby* mutant displayed shorter leaf length^19^. *Kan2* (XM_011029815.1) was OLI, and the double mutation of *kan1* and *kan2* caused narrow leaves^2^. These 3 PHMA mRNAs might be responsible for the changes in leaf shape from Li to Bo. PHMA RNAs can regulate cell proliferation and division by various pathways, *i*.*e*., the auxin, cyclin and cytokinin signalling pathways. For example, auxin influences cell division and cell elongation and has a major impact on the final shapes and functions of cells and tissues in all higher plants^20^. In this study, 31 PHMA mRNAs were involved in the auxin-activated signalling pathway; among them, 20 genes were CLI, and 11 genes were OLI (D1). These results suggested that the activity of the auxin signalling pathway decreased from Li to Bo leaves and that the auxin content in leaves gradually decreased from juvenile trees to adult trees^21^. Generally, cell growth and cell expansion play pivotal roles in leaf shape development^15^. Gibberellin can promote cell elongation^22^. In this study, 16 PHMA mRNAs were involved in the gibberellin signalling pathway; 10 of these genes were OLI, while the other 6 genes were CLI (D1). Seven PHMA mRNAs were involved in cell growth and expansion via other pathways, and 5 genes were OLI, while 2 genes were CLI (D1). Lin found many small cells in the palisade tissues and mesophyll tissues in Li, but in Bo, these cells were more uniform^23^. Our results indicated that the regulation of cell growth was gradually reinforced from Li to Bo and that cell growth might play a key role in *P*. *euphratica* heterophyll morphogenesis. Some PHMA RNAs could regulate leaf shape development by indirect pathways. Eight PHMA mRNAs belonged to the *spl* family, and they were all OLI (D1); among them, 5 members of this family, including *spl2* (XM_011022433.1 and XM_011022434.1) and *spl9* (XM_011034944.1), were co-regulated by ptc-miR156a, ptc-miR156g, MH663515 and MH663526 (Fig. 6). In *Arabidopsis*, downregulated miR156 and upregulated *spl2* and *spl9* could cause leaves to become narrower^14,24^, which was inconsistent with our results in *P*. *euphratica*. Considering that the LI changes from juvenile to adult plants for *Arabidopsis* and *P*. *euphratica* were opposite, miR156a, miR156g, MH663515, MH663526 and *spl* may cooperate to affect leaf shape by regulating the vegetative phase transition. All the above results indicated that, from Li to Bo leaves, the cell division process was generally repressed, while cell growth tended to be reinforced. However, these activities were not uniform in the different axes, implying that the regulators of polar establishment and developmental phase could play a key role in these processes.

In conclusion, 1374 mRNAs, 19 miRNAs, 71 lncRNAs and 2 circRNAs were identified as PHMA RNAs. Among them, 46 PHMA mRNAs, 17 miRNAs, 11 lncRNAs and 2 circRNAs interacted with each other. According to the GO and KEGG results, PHMA RNAs were mainly involved in metabolic process, response to stimulus and development. We could summarize that both external environmental factors and genetic factors co-regulated the expression of PHMA RNAs in *P*. *euphratica*. These RNAs caused cell division to be repressed, mainly through regulating metabolic processes, stress response and development, but cell growth was reinforced, eventually resulting in the morphogenesis of *P*. *euphratica* heterophylls.

## Methods

### Plant materials

Second juvenile leaves, including the Li, La, Ov and Bo leaf groups, were sampled from *P*. *euphratica* aged 0–3, 4–6, 8–12 and over 20 years in Alar (81°17′56.52″E, 40°32′36.90″N), Xinjiang province, China, in April 2017, when there were 7 to 13 unfolded leaves in one bud. The 12 samples were referred to as Li1, Li2, Li3, La1, La2, La3, Ov1, Ov2, Ov3, Bo1, Bo2 and Bo3, respectively. These samples were processed following the methods of Zhao and Qin for RNA-seq, miRNA-seq and qPCR^5^. For all juvenile leaves within typical buds in each sample, leaf length and leaf width were also obtained following the methods of Zhao and Qin^5^, and their averages were referred to as the leaf shape index of the sample.

### RNA extraction and strand-specific RNA sequencing

Total RNA was extracted using the mirVana miRNA Isolation Kit (Ambion) following the manufacturer’s protocol. RNA integrity was evaluated using the Agilent 2100 Bioanalyzer (Agilent Technologies, Santa Clara, CA, USA). Samples with RNA Integrity Number (RIN) ≥7 were subjected to the subsequent analysis. Strand-specific libraries (dUTP) were constructed using TruSeq Stranded Total RNA LT - (with Ribo-Zero Plant) according to the manufacturer’s instructions. Then, these libraries were sequenced on the Illumina sequencing platform (HiSeqTM 2500), and 150 bp paired-end reads were generated.

### Identification of mRNAs associated with *P. euphratica* heterophyll morphogenesis

The clean reads obtained from sequencing were compared with the *P. euphratica* genome (GCF_000495115.1_ PopEup_1.0_genomic.fna.gz, https://www.ncbi.nlm.nih.gov/genome/13265) by TopHat2 (−r 50-library-type fr-firststrand)^25^. Then, these clean reads were further compared with the *P*. *euphratica* transcriptome (GCF_000495115.1_PopEup_1.0_rna.fna.gz, https://www.ncbi.nlm.nih.gov/genome/13265) to calculate transcript abundance by Bowtie2 (−k30 −t)^26^ and eXpress (−rf-stranded)^27^, and the calculations were quantified as FPKM (fragments per kb per million reads)^28^. The data normalization and change fold acquisition (basemean) were performed with DESeq (default)^29^, and the significantly differential expression analysis was carried out following the method of Zhao and Qin^5^. The LI continually decreased from Li to Bo leaves (Fig. 1) in *P*. *euphratica*, and the largest difference occurred between Li and Bo leaves (Table 1)^6^. If the expression patterns of mRNAs were CLI or OLI, meaning that these mRNAs were consistently downregulated or upregulated from Li to Bo leaves, and there was a significant difference between Li and Bo, these mRNAs were considered PHMA mRNAs.

### Identification of lncRNAs associated with *P. euphratica* heterophyll morphogenesis

The strand-specific RNA sequencing results of 12 samples were rebuilt based on a probabilistic model using cufflinks (−no-update-check-library-type fr-firststrand)^30^. All transcripts were compared with the *P*. *euphratica* transcriptome to remove known coding transcripts and loci. Then, transcripts shorter than 200 bp or with less than 2 exons were removed. The remaining transcripts were analysed with CPC (default)^31^, CNCI (−m pl)^32^, Pfam (−e_seq 0.001)^33^ and PLEK (default)^34^ to remove coding transcripts and obtain the lncRNAs of *P*. *euphratica*. The expression abundance quantification, differential expression analysis and PHMA lncRNA identification were performed using the same method as for the mRNAs (see above).

### Identification of circRNAs associated with *P. euphratica* heterophyll morphogenesis

Based on the sequencing results of 12 samples, circRNAs were predicted de novo with CIRI (command: CIRI_v2.0.1pl-Ialn-pe.sam-O CircRNA.gtf-S 100000-F genome.fa-M Mt-A reference.gtf)^35^, and the circRNA sets were obtained. Their expressive abundance in every sample was quantified as RPM (spliced reads per million)^35^. Differential expression analysis and PHMA circRNA identification were also performed using the same method as for the mRNAs (see above).

### MicroRNA sequencing and bioinformatics analysis

MicroRNA sequencing and raw data/reads processing followed the methods of Zhao and Qin^5^ by Illumina analysis (OE Biotech, Shanghai, China). Known miRNAs were identified by aligning against the miRBase v.21 database (http://www.mirbase.org/)^36^ (mismatch = 0). Unannotated small RNAs were analysed by miRDeep2 (−c −j −l 18 −m)^37^ to predict novel miRNAs. Based on the hairpin structure of the pre-miRNA and the miRBase database, the corresponding miRNA star sequence was also identified.

### Identification of miRNAs associated with *P. euphratica* heterophyll morphogenesis

miRNAs were quantified and normalized to TPM (transcripts per million)^8^. The differential expression analysis and PHMA miRNA identification were also performed using the same method as for the mRNAs (see above).

### qPCR analysis

The expression profiles of 4 randomly selected RNAs, including the mRNA XM_011028982.1, miRNA MH663522, lncRNA XR_843201.1 and circRNA MH663520, were verified by qPCR, and 18S RNA was used as an internal reference. To determine the resistance of circRNAs to RNase R digestion, RNase-resistance experiments for MH663520 and MH663526 were further performed following the methods of Li *et al*.^38^ RNase R (Guangzhou Geneseed Biotech Co. Ltd), qPCR kit (Takara, Dalian, China) and TB Green^TM^ Premix Ex Taq^TM^ (Takara, Dalian, China) were used in this experiment according to the manufacturers’ protocols. The Li, La, Ov and Bo leaves were independently analysed both for RNase R resistance and by qPCR. The qPCR system was as follows: 10 µL of TB Green^TM^ Premix Ex Taq^TM^, 2 µL of cDNA, 0.8 µL each of the upstream and downstream primers, and 6.4 µL of RNase-free ddH_2_O. qPCR was carried out with Bio-Rad conditions: denaturation at 95 °C for 30 s, followed by 40 cycles at 95 °C for 5 s, 59 °C for 30 s, and 72 °C for 60 s.

### Interaction analysis between different RNAs and network construction

The interactions of miRNAs and their targets were determined by two steps. First, miRNA-mRNA pairs were identified using psRNATarget (default)^39^. Second, by coupling their expression profiles with the above results, an interaction analysis of the miRNAs and mRNAs was performed. If the expression patterns of a miRNA and its targets were opposite, interactions were regarded as occurring.

Interactions between lncRNAs or circRNAs and their targets were determined by two steps according to the ceRNA hypothesis. First, lncRNA-mRNA or circRNA-mRNA pairs were identified based on having the same miRNA response elements by psRNATarget^33^. Second, by coupling their expression profiles with the above results, if the expression patterns of lncRNAs or circRNAs and mRNAs that shared the same miRNAs with them were similar, then interactions were also regarded as occurring.

### Functional predictions of RNAs

The sequences of PHMA mRNAs were compared with the transcriptome of *Arabidopsis* (GCF_000001735.3_TAIR10_rna.fna.gz, https://www.ncbi.nlm.nih. gov/genome/?term=arabidopsis+thaliana) to obtain the homologous genes of these mRNAs in *Arabidopsis*. Finally, the symbols of these homologous genes were submitted to GO^10^ and KEGG^40,41^ pathway analysis. The functions of the PHMA mRNAs were obtained directly, but the functions of PHMA miRNAs, lncRNAs and circRNAs were predicted based on the functions of their targets.

## Electronic supplementary material

Supplementary Dataset

## Data Availability

All raw RNA-seq and small RNA sequencing data will be available in GEO under the accession numbers GSE120822 (total), GSE120818 (RNA-seq), and GSE120821 (miRNA-seq) after Oct. 2019 or are available from the corresponding author on reasonable request now.
